# Speckle Noise Removal in Image-based Detection of Refractive Index Changes in Porous Silicon Microarrays

**DOI:** 10.1038/s41598-019-51435-y

**Published:** 2019-10-18

**Authors:** Ruyong Ren, Zhiqing Guo, Zhenhong Jia, Jie Yang, Nikola K. Kasabov, Chuanxi Li

**Affiliations:** 1 College of Information Science and Engineering, Xinjiang University, Urumqi, 830046 China; 2 Institute of Image Processing and Pattern Recognition, Shanghai Jiao Tong University, Shanghai, 200240 China; 3 Knowledge Engineering and Discovery Research Institute, Auckland University of Technology, Auckland, 1020 New Zealand; 4 School of Physical Science and Technology, Xinjiang University, Urumqi, 830046 China

**Keywords:** Optics and photonics, Optical physics

## Abstract

Based on porous silicon (PSi) microarray images, we propose a new method called the phagocytosis algorithm (PGY) for removing the influence of speckle noise on image gray values. In a theoretical analysis, speckle noise of different intensities is added to images, and a suitable denoising method is developed to restore the image gray level. This method can be used to reduce the influence of speckle noise on the gray values of PSi microarray images to improve the accuracy of detection and increase detection sensitivity. In experiments, the method is applied to detect refractive index changes in PSi microcavity images, and a good linear relationship between the gray level change and the refractive index change is obtained. In addition, the algorithm is applied to a PSi microarray image, and good results are obtained.

## Introduction

Biochips (microarrays) are a very important technology in the field of life science. Because of their excellent characteristics, extensive application prospects and rapid development, biochips have demonstrated great application value in disease diagnosis, drug development, genetic modification, allergen detection, and environmental protection since they were first proposed in the 1990s. At present, in some biochip-based detection methods, the target molecules or probe molecules are marked with fluorescent markers^1–3^. However, fluorescent markers are expensive, resulting in high costs. Additionally, the introduction of fluorescent markers may alter the structure and activity of biomolecules, thus affecting the test results^4^. For these reasons, label-free detection technology has gradually become an important direction of development in the field of biological analysis^5–18^.

Recently, a highly sensitive, low-cost, label-free biological detection method based on a porous silicon (PSi) microarray has been proposed^19,20^. In this new type of PSi microarray, each cell consists of a one-dimensional photonic crystal with defect layers (microcavities)^20^. If a biological reaction occurs in a microcavity, its refractive index will increase, causing the defect-state wavelength to exhibit a redshift^19^. Consequently, the reflectivity with respect to an incident laser tuned to the defect-state wavelength will increase. This process is equivalent to an increase in the refractive index of the PSi microcavity for laser light at vertical incidence^21^. Through calculations and experiments, it has been proven that the change in the average gray level of a cell in an image of such an array is proportional to the change in the refractive index^19^. Therefore, the corresponding change in the refractive index can be determined from the change in the image gray level. This method has a high detection sensitivity and can be used to detect refractive index changes of less than 10^−4^ ^19^. Thus, image-based analysis of a PSi microarray can be used to quantitatively analyze the biological response of each cell in the microarray by means of high-sensitivity refractive index detection based on the gray levels of the cells. This spectroscopy-free detection method can be performed in a parallel configuration.

Early on, several methods of analyzing PSi microarray images were proposed. Based on digital image processing, these methods could automatically extract the test areas from PSi microarray images^22^.

Due to the rough surface of PSi, interference among the scattered light rays under laser light illumination results in the formation of speckle noise on the photosensitive surface of a CCD^23^. This noise not only reduces the resolution and contrast of the image^24^ but also causes changes in the image gray levels. The occurrence of biological reactions in each cell of a PSi microarray is measured via the parallel detection of the average image gray level of each cell in the array. Before the biological reaction, each cell of the PSi array is modified, and probe biomolecules (such as DNA or antibodies) are immobilized in the PSi. After the reaction, the reactants formed by the specific binding of the target biological molecules (such as complementary DNA or antigens) and the probe molecules are also fixed in the PSi. This results in different morphologies of the PSi surface before and after the biological reaction, resulting in different speckle noise intensities in the corresponding images. In addition, the concentrations of the target biomolecules are different, and the amounts of reactants formed are different, also leading to different levels of speckle noise in the images. These different speckle noise intensities will cause different amounts of interference with the gray levels of the images, thus severely affecting the detection accuracy. To address this problem, the influence of speckle noise on the image gray levels is analyzed. A method of reducing the influence of speckle noise on the gray levels of PSi microarray images is proposed, and this method is demonstrated to improve the accuracy of the detection results.

## Theoretical Analysis

### Speckle noise model

In PSi microarray images, speckle noise is caused by the interaction between the laser light and the roughness of the PSi surface. The existence of speckle noise is inherent to this detection method, and it exhibits distinctive characteristics in terms of its certainty and randomness. Speckle noise can be modeled as multiplicative noise, with the obtained signal being a product of the original signal and the speckle noise^25^. Let I(i, j) denote a distorted pixel in an image, and let M(i, j) denote the corresponding noiseless image pixel. According to the multiplicative noise model,

1 $${\rm{I}}({\rm{i}},{\rm{j}})={\rm{M}}({\rm{i}},{\rm{j}})\times {\rm{N}}({\rm{i}},{\rm{j}})$$

where N(i, j) represents the speckle noise signal^25^.

**Figure 1:**
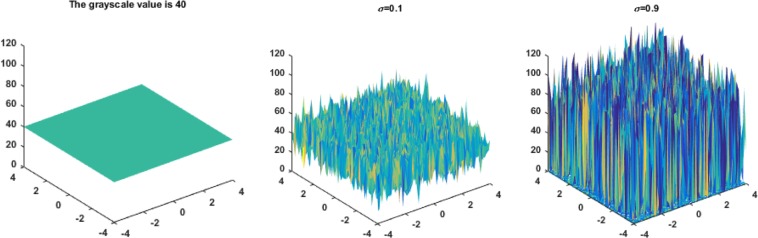
The effects of speckle noise with variances of 0.1 and 0.9 on image gray values.

Speckle noise also affects the gray values of the image itself. The larger the variance of the speckle noise is, the greater its influence on the gray values of the image, and the more difficult it is to recover the noiseless image. To better explain the influence of speckle noise, the effects of speckle noise with variances of 0.1 and 0.9 on an image with a gray value of 40 are shown in Fig. 1.

If the mean values of the N normal speckles participating in the superposition are denoted by $\overline{Ik}$, K = 1, 2, …, N, then the probability density function of the intensity of the kth normal speckle, *Ik*, is an exponential distribution^26^, as shown in formula (2):

2 $${P}_{{I}_{k}}({I}_{k})=\{\begin{array}{cc}\frac{1}{\overline{{I}_{k}}}\exp (-\frac{{I}_{k}}{{I}_{k}}) & {I}_{k}\ge 0\\ 0 & {I}_{k} < 0\end{array}$$

Because the *I*_*k*_ are statistically independent, the probability density function of their sum can be calculated as N-1 reconvolutions of the corresponding functions *P*_*I*_*(I)*, as shown in formula (3):

3 $${P}_{I}(I)={P}_{{I}_{1}}(I)\otimes {P}_{I2}(I)\otimes \cdot \cdot \cdot \otimes {P}_{{I}_{k}}(I)$$

If the N *I*_*k*_ are not zero and are not equal to each other, then formula (2) can be rewritten as follows:

4 $${P}_{I}(I)=\{\begin{array}{cc}\frac{{I}^{(N-1)}}{\Gamma (N){{I}_{0}}^{N}}\exp (-\frac{I}{{I}_{0}}) & I\ge 0\\ 0 & I < 0\end{array}$$

**Figure 2:**
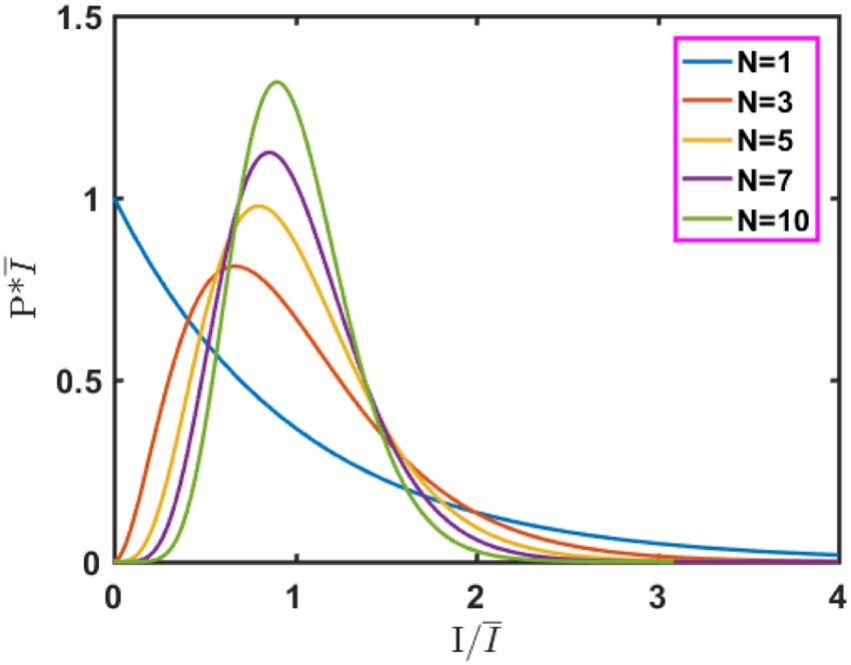
Probability density function of the sum of N independent equal-intensity speckle patterns.

The probability density functions of N different independent speckle patterns of equal intensity are shown in Fig. 2. As N changes with the average total strength remaining unchanged, the probability density function changes from a negative exponential distribution (N = 1) to a Gaussian density function distribution (N = 10), which is consistent with the central limit theorem.

### Influence of speckle noise

**Figure 3:**
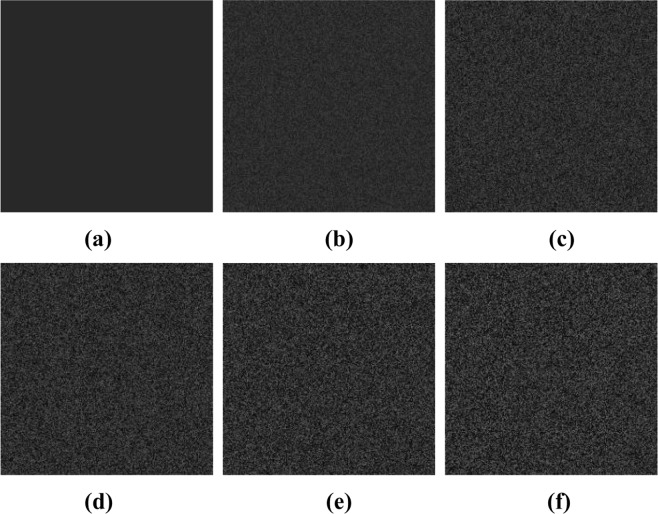
Experimental images generated by MATLAB: (**a**) original image; (**b**) Var = 0.1; (**c**) Var = 0.3; (**d**) Var = 0.5; (**e**) Var = 0.7; (**f**) Var = 0.9.

Figure 3a shows an original image with a gray level of 40 at each pixel. Speckle noise of different intensities (variances) was added to this original image in MATLAB (2016a, MathWorks, Natick, MA, USA) to generate noisy experimental images. The variance (Var) of the noise was varied between 0 and 1 in intervals of 0.1; thus, 10 images with different speckle intensities were generated. The corresponding variances are 0.1, 0.2, 0.3, 0.4, 0.5, 0.6, 0.7, 0.8, 0.9, and 1.0. In Fig. 3b–f, five of the resulting noisy images are presented.

In research on speckle noise, previous works have evaluated denoising algorithms in two ways. Subjective evaluations concern how the textural details and visual effect of an image are improved after denoising. Objective evaluations rely on quantitative metrics such as the speckle index (SI), peak signal-to-noise ratio (PSNR), equivalent number of looks (ENL), and standard deviation (SD).

**Figure 4:**
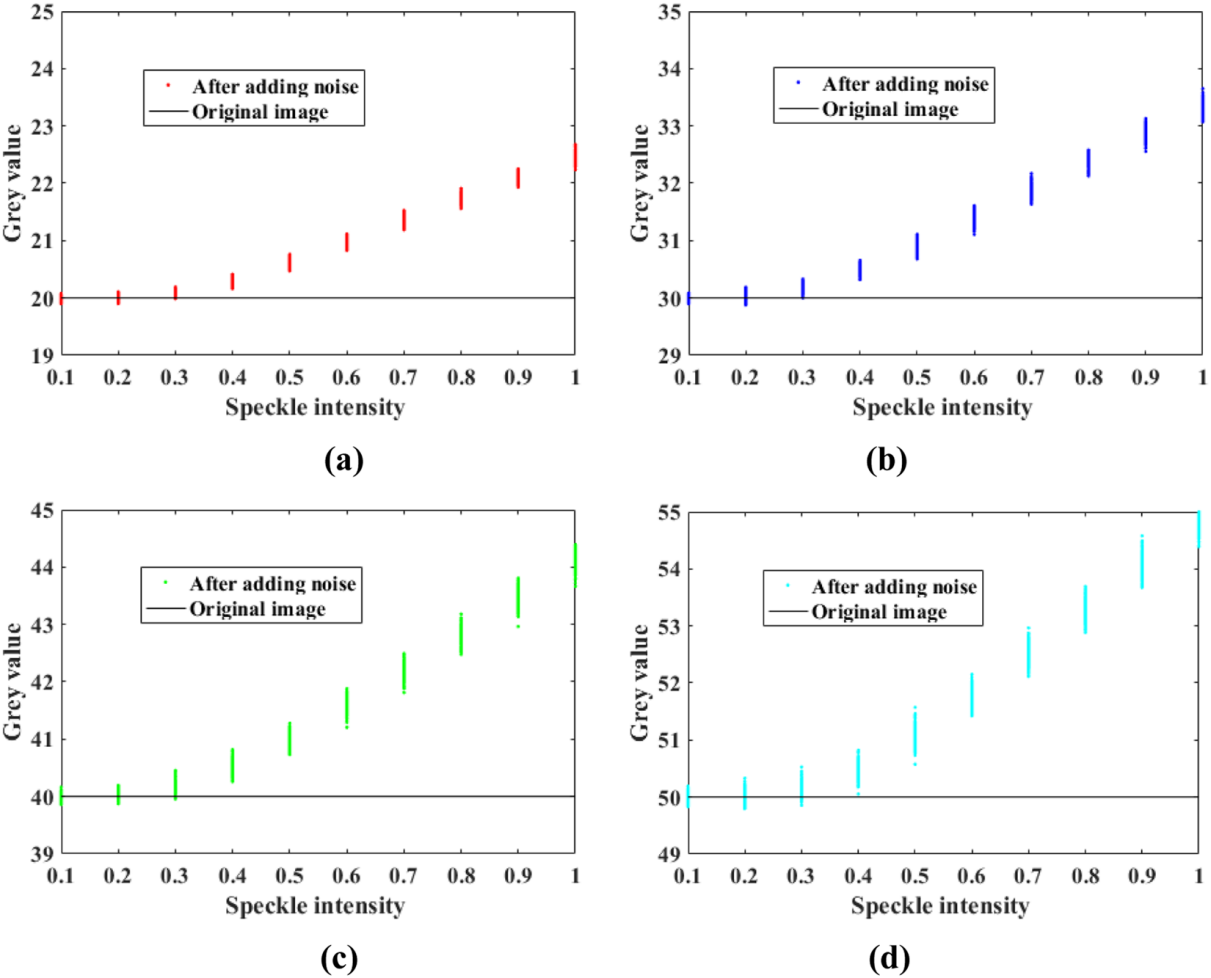
Influence of speckle noise on image gray values: (**a**) gray value of 20; (**b**) gray value of 30; (**c**) gray value of 40; (**d**) gray value of 50.

For the denoising of PSi microarray images, however, the subjective effects and traditional objective indicators are not important. Because the final detection result depends only on the average gray level of each cell in the array, these gray levels need to be restored to remove the influence of the noise to enable accurate detection. At present, there are no related works on the restoration of gray levels affected by speckle noise. To study the effects of speckle noise on the image gray values, we added speckle noise with variances from 0 to 1 to 2500 images with a gray value of 40 and obtained the corresponding relationship between the resulting average gray value and the speckle intensity, as shown in Fig. 4c. As the speckle intensity increases, the average gray value of the image increases. The same result was found after testing testing three other sets of 2500 images each, with initial gray values of 20, 30 and 50.

**Figure 5:**
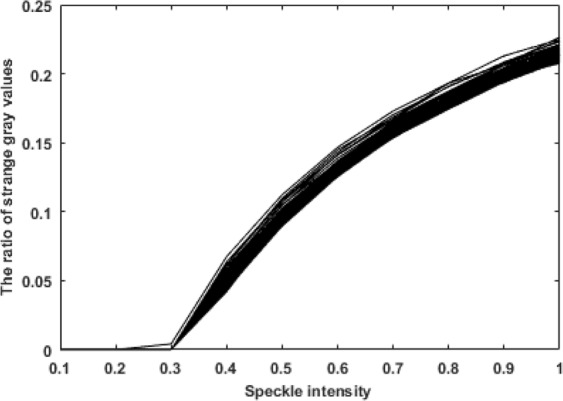
Relationship between the speckle intensity and the proportion of singular pixels.

To explain the reason for the observed relationship between the speckle intensity and the average gray value, we analyzed 7500 images containing speckle noise and considered the speckle intensity and the number of singular pixels (bright spots with an abnormal increase in gray value). The relationship between the speckle intensity and the number of singular pixels is shown in Fig. 5. By comparing Figs 4 and 5, it can be seen that the variation in the average gray value of the image is related to the number of singular pixels. When the speckle intensity is within the range of [0, 0.3], the number of bright spots is very small. Hence, the randomness of the scattered noise becomes the main factor affecting the average gray value of the image, causing the average gray value to fluctuate around the original gray value. As the speckle intensity increases beyond 0.3, the number of bright spots gradually increases, and these bright spots become the main factor affecting the average gray value of the image, resulting in an overall increase in the average gray value.

The findings from Fig. 5 can be used as follows:

1. The results provide a basis for the new algorithm proposed below:
2. An approximate relationship between the speckle intensity and the number of singular points can be obtained. By analyzing an unknown grayscale image, it is possible to estimate the degree of speckle contamination of the image, which will then play a large role in the subsequent image analysis.

The above conclusions are very important for the detection of biological reactions in PSi microarrays based on digital images^19–21^. In this image-based detection method, the changes in refractive index that are caused by biological reactions are determined by measuring the changes in the gray values of PSi microarray images. Speckle noise produced by laser irradiation will always be present in PSi microarray images obtained directly with digital imaging equipment. Moreover, the speckle noise intensities will be different for PSi surface images obtained before and after a reaction. According to the findings reported above, the higher the speckle noise intensity is, the greater its impact on the image gray levels. Thus, the changes in the gray levels measured in a biological detection experiment are partly due to the true changes in the refractive index of the PSi before and after the reactions and partly due to image speckle noise. Therefore, the ability to remove speckle noise in PSi microarray images is very important for the detection of biological reactions.

## Methods

Many excellent algorithms have emerged from research on speckle noise. The classical algorithms include common Lee filters, Kuan filters, mean filters, and median filters^27–29^. Previous works have introduced some improvements to these classical algorithms and have achieved good results^30–35^. Recently, scholars have also proposed advanced algorithms, such as probability-based nonlocal means filtering^36^, numerical multilook and 3D block matching filtering^37^, adaptive wavelet threshold processing^38^, and adaptive anisotropic diffusion^39^. In this paper, a variety of filtering algorithms are experimentally analyzed. Most algorithms show good performance with respect to common evaluation indexes, such as the SI, PSNR and ENL, but they cannot modify the gray level of an image. Therefore, these algorithms cannot restore the gray level in the presence of speckle noise. The weights in the BM3D and PNLM algorithms are determined by the distances between similar blocks, and the gray value of a target pixel is obtained by weighting the gray values of all the pixels in its neighborhood. A large number of scattered spots and speckle blocks will increase the gray value differences between two neighborhood windows and thus affect the accuracy of the weights. The gradients of images with scattered spots and speckle blocks will be large, and the good edge retention of the PM algorithm can result in incorrectly identified edges and a poor denoising effect. A large number of scattered spots and speckle blocks will similarly affect the selection of the optimal threshold for the adaptive wavelet transform, thereby worsening the denoising effect. For the classical median and adaptive median algorithms, a large number of scattered spots and speckle blocks will result in a large difference between the selected median pixel and the original pixel, affecting the denoising process. To overcome these challenges, in this paper, we develop a new method of processing images with speckle blocks and a large number of scattered spots: the phagocytosis algorithm (PGY). This method not only can recover images contaminated with speckle noise but also exhibits good stability.

**Figure 6:**
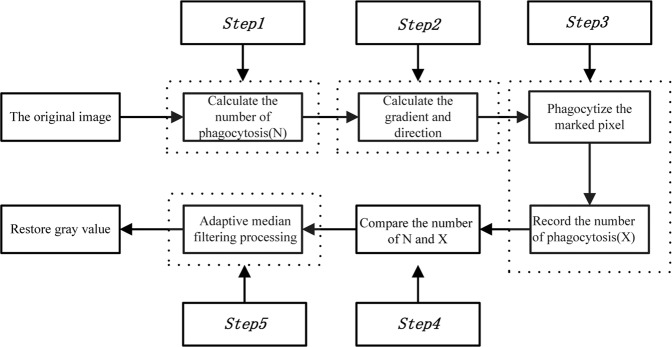
Algorithm flow chart.

The flow chart of the phagocytosis algorithm used in this paper is shown in Fig. 6. The specific steps are described below.

**Figure 7:**
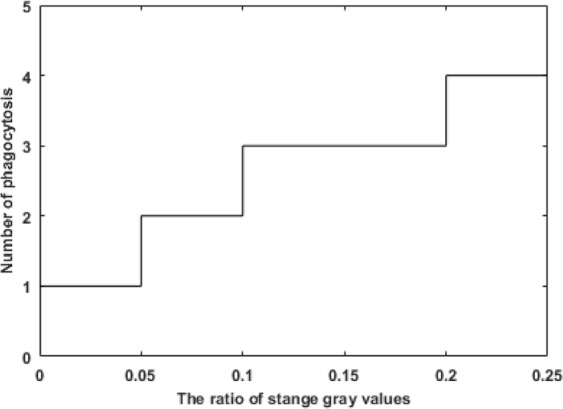
Relationship between the proportion of singular pixels and the number of phagocytosis iterations.

Note that there exists an optimal number of phagocytosis iterations: an excessively large number of iterations will greatly impair the time performance of the algorithm, while an insufficient number of iterations will result in an inability to remove the speckle noise. In this study, 17500 speckle images in different formats and with different gray values were experimentally investigated to determine the optimal number of phagocytosis iterations. As a result, the relationship between the optimal number of phagocytosis iterations and the proportion of singular pixels was determined as shown in Fig. 7.

Step 1: The proportion of singular pixels is calculated for an unknown gray image, and the optimal number of phagocytosis steps (N) is found according to Fig. 7.

Step 2: The gradient magnitudes and directions for 8 neighborhoods in the smoothed gray image are calculated using a 3 × 3 template. Thresholds are applied to identify not only the speckle points but also the edges of speckle blocks based on the first-order partial derivatives at each pixel, which are calculated as follows.

Partial derivative in the X direction:

5 $${G}_{x}(x,y)=S(x+1,y)-S(x-1,y)$$

Partial derivatives in the Y direction:

6 $${G}_{y}(x,y)=S(x,y+1)-S(x,y-1)$$

Partial derivatives in the 45° direction:

7 $${G}_{{45}^{\circ }}(x,y)=S(x-1,y+1)-S(x+1,y-1)$$

Partial derivatives in the 135° direction:

8 $${G}_{{135}^{\circ }}(x,y)=S(x+1,y+1)-S(x-1,y-1)$$

The expression of gradient amplitude calculated by second-order Euclidean norm is as follows:

9 $$G(x,y)=\sqrt{{G}_{x}{(x,y)}^{2}+{G}_{y}{(x,y)}^{2}+{G}_{{45}^{\circ }}{(x,y)}^{2}+{G}_{{135}^{\circ }}{(x,y)}^{2}}$$

The calculation expression of gradient direction is as follows:

10 $$\theta (x,y)={\tan }^{-1}({G}_{y}(x,y)/{G}_{x}(x,y))$$

Here, *G*_*x*_*(x, y)* is the gradient at point (x, y) in the X direction, *G*_*y*_*(x, y)* is the gradient at point (x, y) in the Y direction, *G*_*45°*_*(x, y)* is the gradient at point (x, y) in the 45° direction, and *G*_*135°*_*(x, y)* is the gradient at point (x, y) in the 135° direction.

Step 3: The remaining pixels are divided into 9 regions. The average gray values of the 9 regions are obtained and sorted to obtain the median gray value. The gray value of the identified speckle pixel is replaced with this median gray value. Thus, a phagocytosis iteration is completed, and the iteration number (X) is recorded.

Step 4: Steps (2) and (3) are repeated a number of times equal to the specified number of phagocytosis iterations.

Step 5: The image is further processed with an adaptive median filtering algorithm to remove any remaining speckles.

### Comparison with other methods

**Figure 8:**
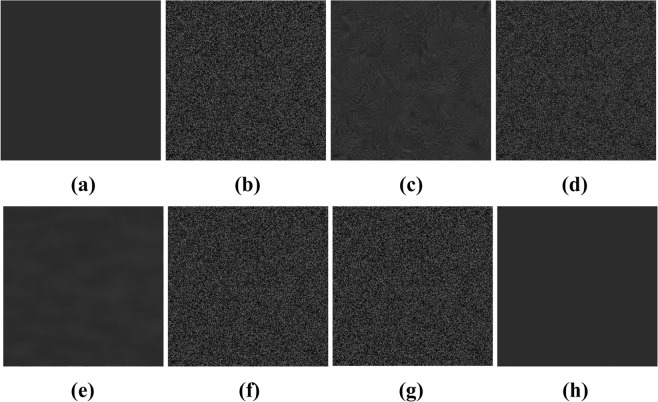
Six algorithm recovery effects. (**a**) Original image; (**b**) After adding noise; (**c**) BM3D; (**d**) Lee; (**e**) PNLM; (**f**) NAWT; (**g**) PM; (**h**) PGY.

Five advanced algorithms previously proposed by other scholars, as implemented in MATLAB, were considered for comparison in terms of calculating the average gray value and SI: probability-based nonlocal mean filtering^36^, numerical multilook and 3D block matching filtering^37^, adaptive wavelet threshold processing^38^, adaptive anisotropic diffusion^39^, and Lee filtering. The average gray values were processed to two decimal places, and the SI was processed to four decimal places. In addition, with regard to the speckle intensity, we used 10 images to calculate the root-mean-square error (RMSE) of the method; the results can well reflect the measurement accuracy, as shown in Table 1. The gray level of an image with a speckle variance of 0.9 was restored, as shown in Fig. 8.

**Table 1:** Data statistics of ten pictures.

Speckleintensity	Index	Unproessed image	Lee	BM3D	NAWT	PNLM	PM	PGY	RMSE
0.10	AGL	40.03	40.19	40.03	40.02	40.04	40.03	40.00	0.0341
	SI	0.3236	0.0470	0.0263	0.0593	0.0364	0.3233	0.0018	
0.20	AGL	40.01	40.12	40.00	40.01	39.94	40.00	40.00	0.0400
	SI	0.4792	0.2520	0.0354	0.0658	0.0234	0.4800	0.0034	
0.30	AGL	40.25	40.28	40.25	40.05	39.95	40.24	40.00	0.0410
	SI	0.5784	0.3880	0.0443	0.0789	0.0297	0.5778	0.0020	
0.40	AGL	40.63	40.69	40.34	40.23	39.98	40.63	40.00	0.0349
	SI	0.6431	0.4708	0.0572	0.0846	0.0340	0.6431	0.0029	
0.50	AGL	41.11	41.04	40.42	40.72	40.03	41‥12	40.00	0.0352
	SI	0.6905	0.5300	0.0795	0.0903	0.0349	0.6885	0.0020	
0.60	AGL	41.53	41.59	40.90	41.03	39.83	41.53	40.00	0.0410
	SI	0.7249	0.5718	0.1127	0.0963	0.0424	0.7276	0.0052	
0.70	AGL	42.10	42.11	40.61	41.40	39.96	42.10	40.00	0.0419
	SI	0.7560	0.6089	0.1522	0.1012	0.0515	0.7545	0.0040	
0.80	AGL	43.03	42.76	41.43	41.93	40.60	43.03	40.00	0.0428
	SI	0.7779	0.6348	0.1931	0.1027	0.0444	0.7734	0.0052	
0.90	AGL	43.47	43.37	41.27	42.17	40.51	43.47	39.99	0.0430
	SI	0.7960	0.6560	0.2321	0.1048	0.0484	0.7957	0.0090	
1.00	AGL	44.00	44.11	41.60	42.20	40.68	44.00	40.00	0.0437
	SI	0.8120	0.6746	0.2741	0.1068	0.0434	0.8146	0.0120

**Figure 9:**
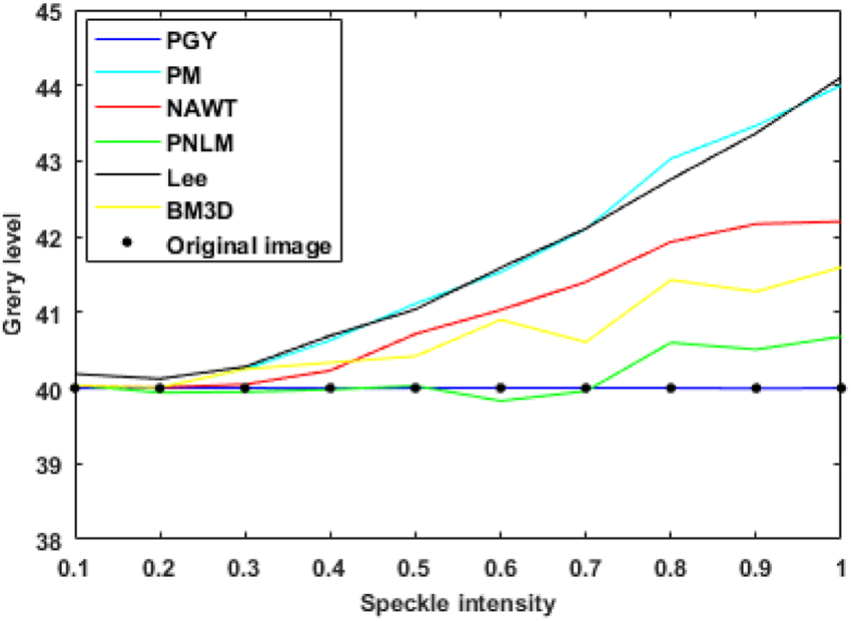
Comparison of the ability of the six algorithms to restore the gray level of an image.

Based on the statistical data from the experimental images, the curves showing the variation in the average gray value with the speckle intensity after the application of the six algorithms were obtained, as shown in Fig. 9.

**Figure 10:**
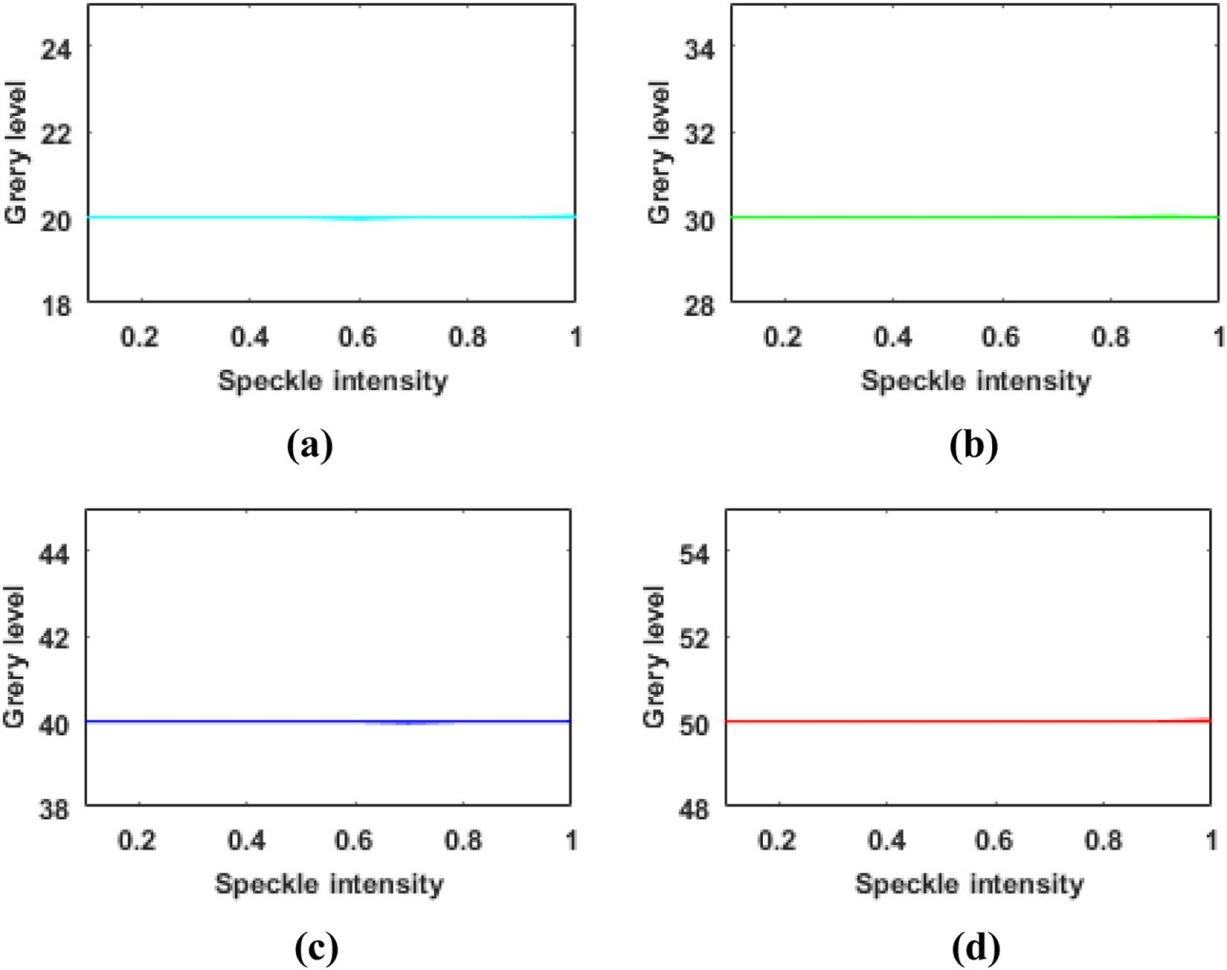
Recovery of different gray values: (**a**) gray value of 20; (**b**) gray value of 30; (**c**) gray value of 40; (**d**) gray value of 50.

As shown in Figs 8 and 9 and Table 1, the phagocytosis algorithm can restore an image contaminated with speckle noise, achieving an SI value close to zero, indicating that the speckle noise has been suppressed. Compared with the five previously proposed algorithms, the phagocytosis algorithm shows a remarkable advantage in terms of recovery ability. To further verify the universality of the phagocytosis algorithm, a restoration test was carried out on 17500 images containing speckle noise in different formats and with different gray values, and the recovery accuracy was 99.97%. Thus, it is shown that speckle noise can be effectively removed from images in different formats and with different gray values. For convenience and clarity, the results for only 2000 speckle-noise-contaminated images with different gray levels are shown in Fig. 10.

### Experimental study

In our previous study, when laser light was incident on the surface of a PSi microcavity at 0° (vertical incidence), the reflection spectrum of the PSi microcavity obtained by increasing the incidence angle was the same as that obtained by increasing the refractive index^21^. Therefore, a change in the refractive index of a PSi microcavity can be considered equivalent to a change in the laser incidence angle. For a small angle of incidence, the enhancement of the reflected light intensity on the microcavity surface is proportional to the increase in the incidence angle^19^, that is, the gray value of a digital image of the microcavity surface increases with an increasing incidence angle^19^. Here, we applied the proposed phagocytosis algorithm to detect refractive index changes in PSi microcavity images. Each microcavity unit is a circular cell region in the PSi microarray.

**Figure 11:**
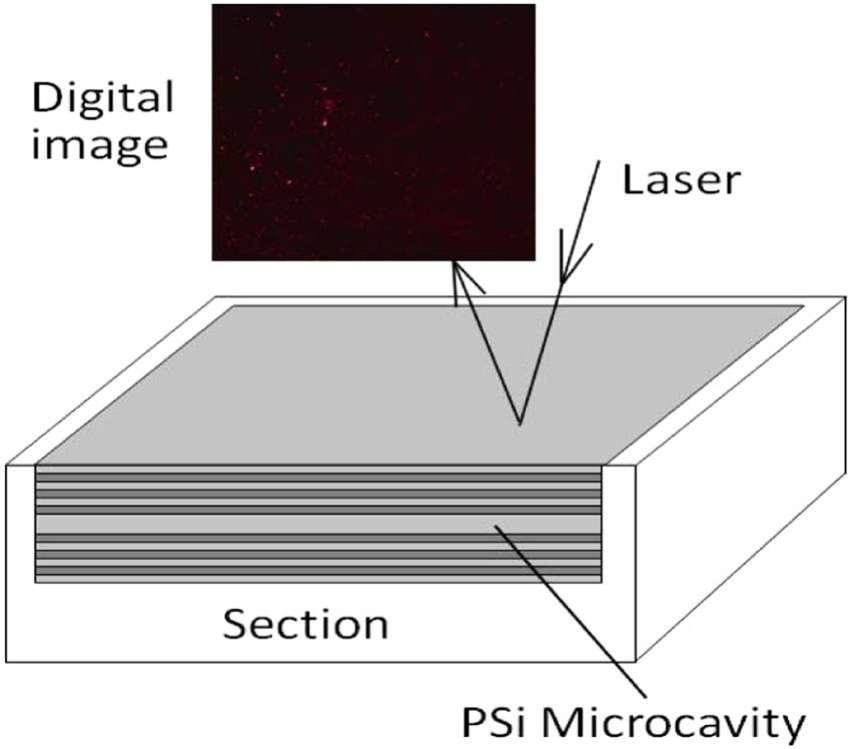
Digital image of light reflected from the surface of a PSi microcavity.

PSi microcavities were prepared via electrochemical etching. Each microcavity consisted of a defect layer sandwiched between two Bragg layers, an upper layer and a lower layer. The Bragg structures on both sides each contained 6 cycles. The refractive index and thickness of each layer in a PSi microcavity are controlled by the corrosion current density and time. The PSi microcavities in our experiment had a defect-state wavelength of 633 nm. The structure of a PSi microcavity is illustrated in Fig. 11.

**Figure 12:**
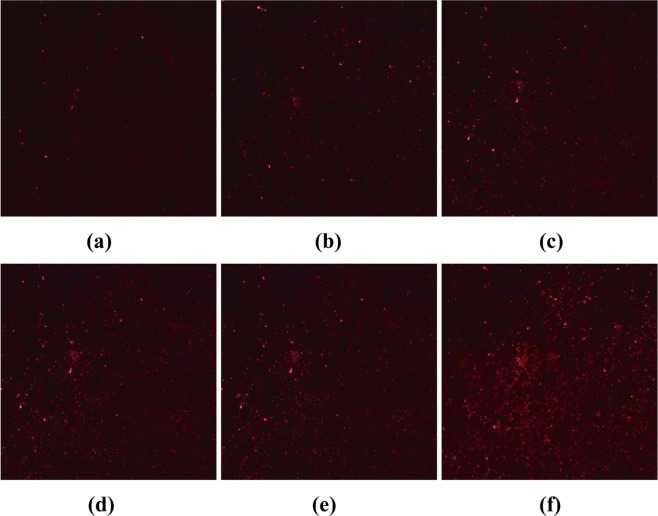
Reflected images of PSi microcavities at different incident angles. (**a**) 0°, (**b**) 1°, (**c**) 2°, (**d**) 3°, (**e**) 4°, (**f**) 5°.

In the detection device^19^, laser light with a wavelength of 633 nm was incident on the PSi microcavities at different angles, and images of the device surface were obtained with a digital imaging device, as shown in Fig. 12.

**Figure 13:**
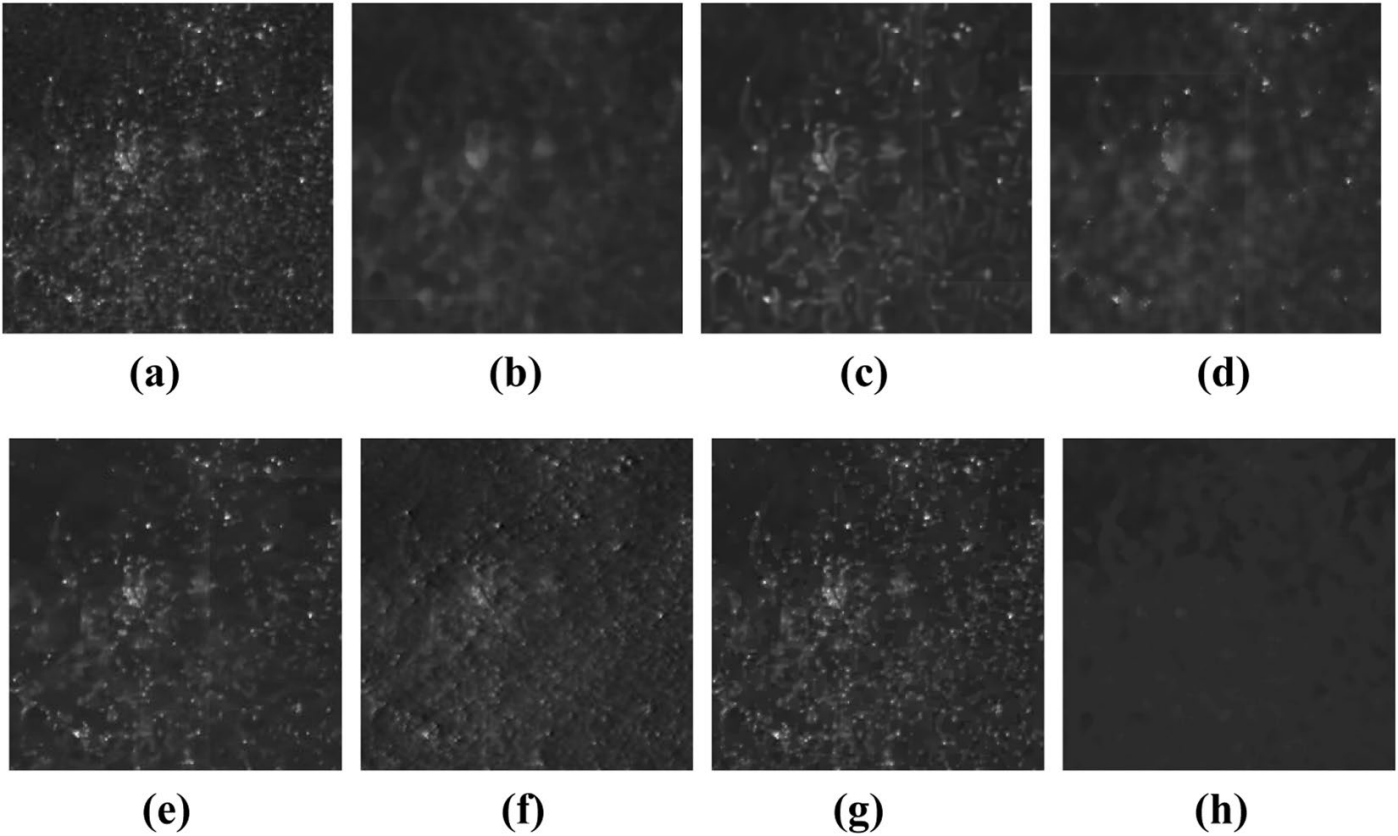
The restoration of gray value of porous silicon microcavity with incident angle of 5°. (**a**) Original image; (**b**) Adapitive median filter; (**c**) BM3D; (**d**) Lee; (**e**) PNLM; (**f**) NAWT; (**g**) PM; (**h**) PGY.

First, the RGB image of the light reflected by the PSi microcavity at an incidence angle of 5° in Fig. 12 was transformed into a grayscale image, and seven methods were used to restore the gray level, as shown in Fig. 13. It can be seen from this figure that the phagocytosis algorithm is effective for eliminating speckles that obscure the real image, can suppress the influence of speckle noise, and can restore the gray value of the image. We compare the results of our proposed algorithm to the results of five other advanced algorithms: probability-based nonlocal mean filtering^36^, numerical multilook and 3D block matching filtering^37^, adaptive wavelet threshold processing^38^, adaptive anisotropic diffusion^39^, and Lee filtering. We find that the adaptive median filtering algorithm does not achieve the desired effect in removing real speckles. Additionally, it does not restore the true gray value of the original image.

**Figure 14:**
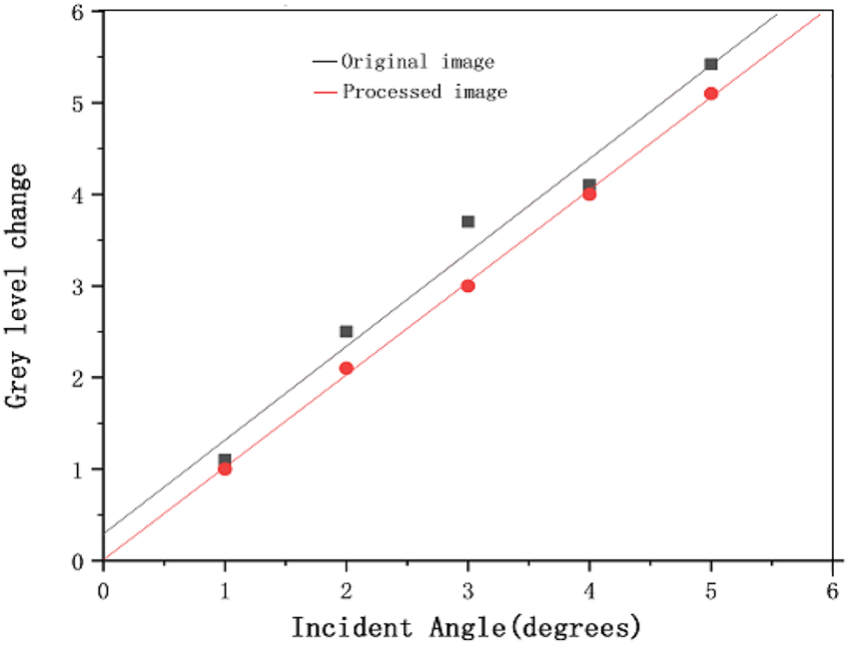
Relationship between the gray value of a PSi microcavity surface image and the laser incidence angle.

Based on the theoretical analysis presented in this paper and the observed effect of real speckle removal, it can be concluded that the phagocytosis algorithm can effectively achieve gray value restoration. Therefore, we applied this algorithm to all images in Fig. 12. The correspondence between the different incidence angles and gray values obtained after processing with the phagocytosis algorithm is shown in Fig. 14.

The fitting formula and judgment coefficient before processing are respectively:

11 $${\rm{Y}}=0.98{\rm{X}}+0.40\,\,{R}^{2}=0.958$$

The fitting formula and judgment coefficient after processing are respectively:

12 $${\rm{Y}}=1.01{\rm{X}}+0.01\,\,{R}^{2}=0.9986$$

It can be seen from Fig. 14 that the linear fit to the data processed with our denoising algorithm is better than the fit to the unprocessed data. In addition, for the same change in gray value, the change in angle as evaluated with our denoising algorithm is larger than that achieved without processing.

**Figure 15:**
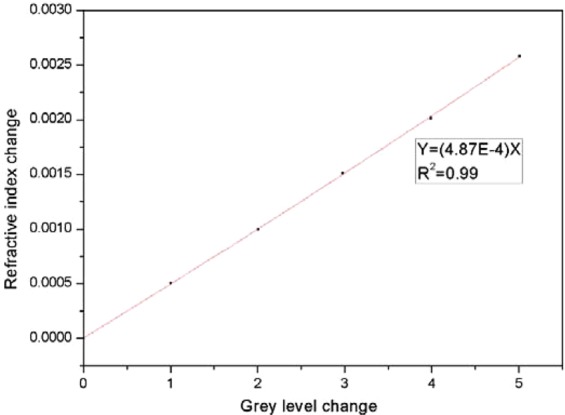
Relationship between the change of refractive index and the change of grey level in PSi microcavity.

From the refractive indexes calculated based on the relationship between the changes in incidence angle and refractive index, the relationship between the changes in the gray level and the refractive index was obtained, as shown in Fig. 15. There is a good linear relationship between the change in the refractive index and the change in the gray level. As seen from Figs 14 and 15, after an image is processed with our denoising algorithm, the obtained change in the refractive index is larger than that obtained before processing for the same change in the gray value, demonstrating that the sensitivity of refractive index detection is improved.

It can be seen from the above experiment that the phagocytosis algorithm is very suitable for recovering the gray levels of images affected by speckle noise. The proposed algorithm can effectively reduce the influence of speckle noise in PSi microarray images and improve the detection accuracy achieved based on those images.

### Gray-level restoration of PSi microcavity array images

**Figure 16:**
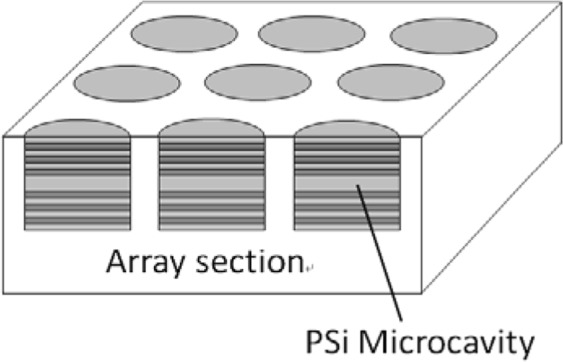
The structures of PSi microcavity array.

A PSi microarray was fabricated on monocrystalline silicon by means of lithography and electrochemical etching technology^19^. Each cell in the array was a microcavity structure, and the device parameters were the same as the microcavity parameters. The structure of the PSi microcavity array is shown in Fig. 16.

**Figure 17:**
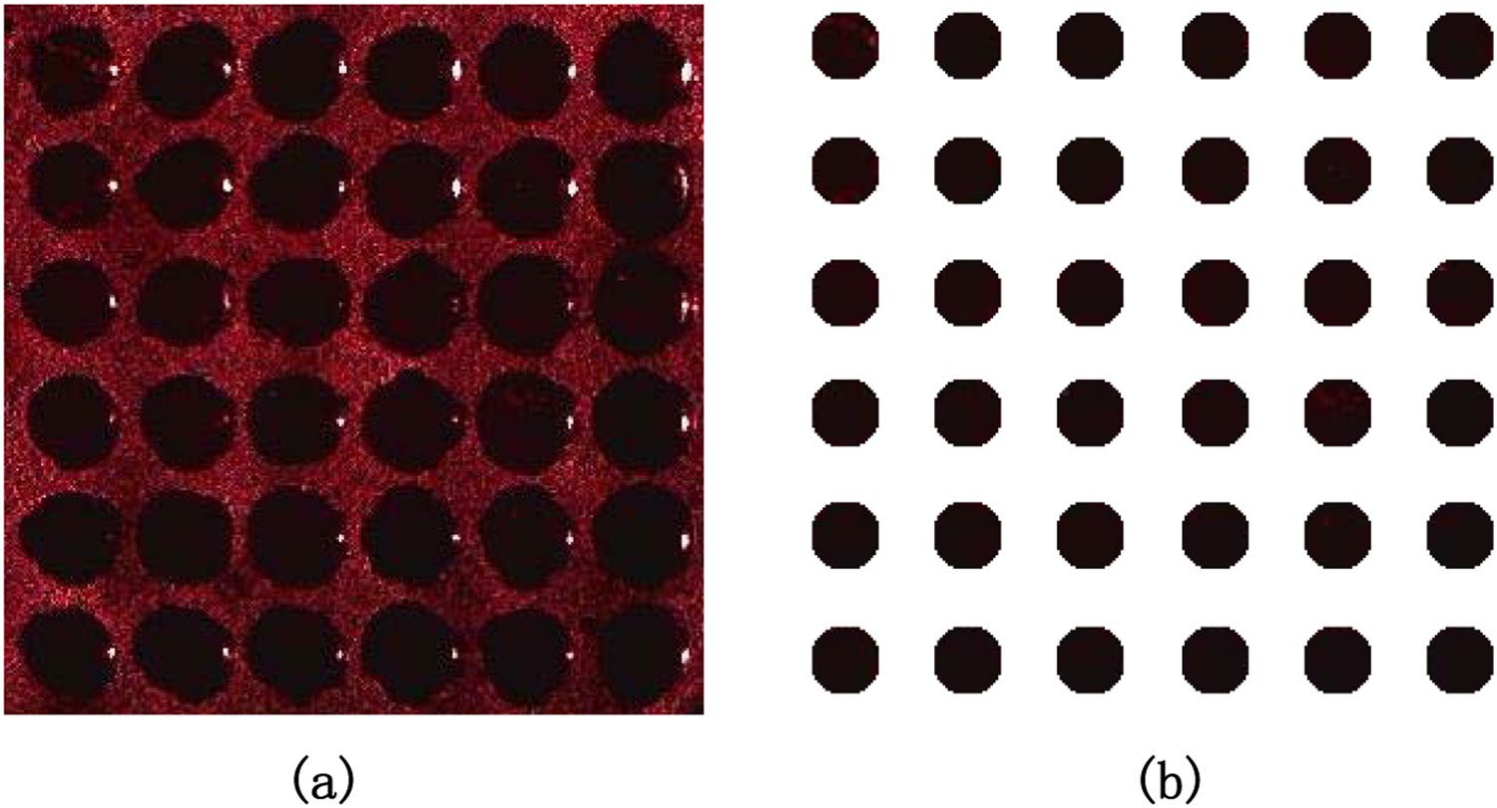
(**a**) Actual PSi microarray image obtained with digital imaging equipment; (**b**) PSi microarray image after pretreatment, correction and segmentation to obtain the sample points to be measured.

Each cell in the array had a defect-state wavelength of 633 nm. Laser light with a wavelength of 633 nm was incident on the surface of the PSi microarray at different angles, and surface images of the array were obtained with a digital microscope. Figure 17a shows a microarray image acquired with an incidence angle of 5°. The theoretical analysis presented above has demonstrated that the phagocytosis algorithm has the best ability to restore the gray levels of such images. Therefore, the proposed algorithm was applied to an actual PSi microarray image to verify this finding. In this experiment, we used previously described methods of preprocessing, tilt correction and sample segmentation^22^. Finally, we obtained the sample points to be measured, as shown in Fig. 17b.

By measuring the average gray level of each sample point in Fig. 17b, we obtained the overall average gray level and the SI of all sample points. The measurement results are shown in Tables 2 and 3. Table 2 shows the measurement results before filtering, and Table 3 shows the measurement results for the sample points after the application of the phagocytosis algorithm.

**Table 2:** The average gray value corresponding to the sample point before filtering.

Column Row	First column	Second column	Third column	Fourth column	Fifth column	Sixth column
First row	17.74	16.00	15.98	16.20	16.03	15.95
Second row	16.28	15.99	16.01	16.06	16.13	15.98
Third row	15.93	16.12	15.99	15.98	15.99	16.25
Fourth row	15.83	15.94	16.12	15.98	16.37	15.99
Fifth row	15.97	15.99	15.95	15.99	16.16	16.03
Sixth row	15.94	15.99	16.00	15.93	15.97	15.93
Average grey level	16.07					
Speckle index	0.5069

**Table 3:** The average gray value corresponding to the sample point after filtering.

Column Row	First column	Second column	Third column	Fourth column	Fifth column	Sixth column
First row	16.01	16.00	16.00	16.00	16.00	16.00
Second row	16.00	16.00	16.00	16.00	16.00	16.00
Third row	16.00	16.00	16.00	16.00	16.00	16.00
Fourth row	16.00	16.00	16.00	16.00	16.01	16.00
Fifth row	16.00	16.00	16.00	16.00	16.00	16.00
Sixth row	15.99	16.00	16.00	15.99	16.00	16.00
Average grey level	16.00					
Speckle index	0.0046					

It can be seen from the above experiment that after filtering with the phagocytosis algorithm, the SI is close to zero, indicating that the gray value can be effectively recovered. The phagocytosis algorithm has a better recovery ability than the other advanced algorithms considered. The proposed algorithm can effectively eliminate the influence of speckle noise and improve the image-based detection accuracy achieved using PSi microarray images.

## Conclusions

This paper reports research conducted on the effect of speckle noise on the gray levels of PSi microarray images. A corresponding theoretical analysis is presented, and an algorithm suitable for restoring image gray levels in the presence of speckle noise is proposed. A PSi microcavity sensor was prepared for an experimental evaluation, and images of the light reflected from the sensor were obtained. The proposed algorithm was applied to detect refractive index changes in the PSi microcavities, and good results were obtained. At present, the algorithm proposed in this paper exhibits better performance than other advanced algorithms in terms of recovering image gray levels in the presence of speckle noise and improving the detection accuracy achieved with a PSi microcavity sensor. There are no related works that have investigated the recovery of image gray levels.
